# Dual transcriptional characterization of spinach and *Peronospora effusa* during resistant and susceptible race-cultivar interactions

**DOI:** 10.1186/s12864-024-10809-x

**Published:** 2024-10-07

**Authors:** Kelley J. Clark, Chunda Feng, Amy G. Anchieta, Allen Van Deynze, James C. Correll, Steven J. Klosterman

**Affiliations:** 1 Department of Agriculture, Agricultural Research Service, Sam Farr United States Crop Improvement and Protection Research Center, Salinas, CA USA; 2 Seed Biotechnology Center, Department of Plant Sciences, University of California-Davis, Davis, CA USA; 3 Department of Agriculture, Agricultural Research Service, Crop Genetics Research Unit, Stoneville, MS USA; 4 Department of Plant Pathology and Entomology, University of Arkansas, Fayetteville, AR USA

**Keywords:** Race-specific, Downy mildew, Spinach, Transcriptomics, Defense, Resistance, Virulence

## Abstract

**Background:**

Spinach downy mildew, caused by the obligate oomycete pathogen, *Peronospora effusa* remains a major concern for spinach production. Disease control is predominantly based on development of resistant spinach cultivars. However, new races and novel isolates of the pathogen continue to emerge and overcome cultivar resistance. Currently there are 20 known races of *P. effusa*. Here we characterized the transcriptomes of spinach, *Spinacia oleracea*, and *P. effusa* during disease progression using the spinach cultivar Viroflay, the near isogenic lines NIL1 and NIL3, and *P. effusa* races, R13 and R19, at 24 h post inoculation and 6 days post inoculation. A total of 54 samples were collected and subjected to sequencing and transcriptomic analysis.

**Results:**

Differentially expressed gene (DEG) analysis in resistant spinach interactions of R13-NIL1 and R19-NIL3 revealed spinach DEGs from protein kinase-like and P-loop containing families, which have roles in plant defense. The homologous plant defense genes included but were not limited to, receptor-like protein kinases (Spiol0281C06495, Spiol06Chr21559 and Spiol06Chr24027), a BAK1 homolog (Spiol0223C05961), genes with leucine rich repeat motifs (Spiol04Chr08771, Spiol04Chr01972, Spiol05Chr26812, Spiol04Chr11049, Spiol0084S08137, Spiol03Chr20299) and ABC-transporters (Spiol02Chr28975, Spiol06Chr22112, Spiol06Chr03998 and Spiol04Chr09723). Additionally, analysis of the expression of eight homologous to previously reported downy mildew resistance genes revealed that some are differentially expressed during resistant reactions but not during susceptible reactions. Examination of *P. effusa* gene expression during infection of susceptible cultivars identified expressed genes present in R19 or R13 including predicted RxLR and Crinkler effector genes that may be responsible for race-specific virulence on NIL1 or NIL3 spinach hosts, respectively.

**Conclusions:**

These findings deliver foundational insight to gene expression in both spinach and *P. effusa* during susceptible and resistant interactions and provide a library of candidate genes for further exploration and functional analysis. Such resources will be beneficial to spinach breeding efforts for disease resistance in addition to better understanding the virulence mechanisms of this obligate pathogen.

**Supplementary Information:**

The online version contains supplementary material available at 10.1186/s12864-024-10809-x.

## Background

Spinach is a popular and nutritious crop belonging to the Amaranthaceae family which also contains beet, chard, and quinoa [1–3]. One of the major disease concerns for spinach remains *Peronospora effusa* which causes downy mildew [4, 5]. Spinach downy mildew renders crops unmarketable due to leaf chlorosis and masses of downy mildew sporangia which appear on the underside of leaves [6]. Consumer-friendly, baby leaf and teen spinach is produced using high density plantings, ranging from about 3–4 million seed per acre, and watered with overhead irrigation creating optimal conditions for downy mildew disease [7, 8]. Management practices are majorly by fungicides and development of resistant spinach cultivars [9–11]. However, for organic spinach production, which represents nearly half of the U.S. market, breeding for pathogen resistance is the major defense strategy.

The number of *P. effusa* races has been increasing rapidly over the past 30 years, with 20 known races as of 2024 [12–19]. The evolution of *P. effusa* races and novel isolates poses a continuous challenge to spinach breeding for resistant cultivars [18]. Moreover, evidence of reticulated evolution and discordance between nuclear and mitochondrial phylogenies indicates sexual reproduction in some isolates of *P. effusa* [19]. The pathogen’s ability to produce sexual oospores, which can remain viable for at least six years [20], likely contributes to genetic variation and subsequent breaking of spinach resistance [19, 20]. Availability of wild spinach gene banks is often limited with majority of accessions being from *S. oleracea* and expeditions to recover new accessions from unexplored regions becoming more difficult [1]. Therefore, there is a need to understand both the race-specificity of the pathogen as well as the cultivar-specific defense mechanisms of the host.

A total of six downy mildew resistance loci RPF1-6 (Resistance to *Peronospora farinosa*) have been identified in spinach [14, 21, 22]. Most commercial spinach cultivars are hybrids which contain a combination of these loci [23]. Near isogenic lines (NILs) carrying these six resistance loci have been developed by backcrossing resistant spinach cultivars to the universally susceptible cultivar, Viroflay. These six NILs, in addition to some commercial cultivars, are used as a differential set to characterize *P. effusa* races and novel isolates [24]. RPF1, 2, and 3 have been mapped to chromosome 3, and some markers exist to select for these resistance alleles [14, 21, 22]. In addition, several resistance genes have been predicted based on their location on RPF1 or 2 and comparative genomic analysis of resistant cultivars [25, 26]. These genes include nucleotide-binding site leucine-rich repeat proteins and receptor kinases [23]. Still, little is known about the molecular functions of these predicted resistance genes or expression during host defense.

There have been recent advances in the genomes of both spinach and *P. effusa* providing improved resources for transcriptional analysis. Hulse-Kemp et al. presented a chromosome-scale genome assembly of the spinach cultivar, Viroflay, which was generated from a combination of Pacific Biosciences and Illumina sequencing reads; providing a genetically anchored assembly with less fragments than previous iterations [27]. From this assembly, 92.5% of the genes have known homologs or functional classifications and 1,004 were predicted as candidate resistance genes. There have also been improvements to the original spinach genome using more modern technology in addition to an additional draft spinach genome [28, 29, 30]. The recently published *P. effusa* genome assembly represents the first telomere-to-telomere genome for an oomycete [31]. The assembly contains 16 nearly gapless chromosomes and 9,745 coding genes, in addition to being larger and containing more repeat regions than previously reported assemblies [32, 33]. A total of 307 *P. effusa* genes were annotated as effectors offering insight to potential virulence mechanisms of the pathogen [31].

Here we expand on a previous study, which surveyed the spinach-*P. effusa* transcriptome of the spinach cultivars Viroflay and Solomon [34], by utilizing two near isogenic lines (NILs) with known resistance to specific *P. effusa* races, in addition to the universally susceptible cultivar, Viroflay. We employed two *P. effusa* races, R19, which is a recently described race with limited commercial resistance available and R13 for its opposite compatibility to the NILs compared to R19. Both races have been observed in major spinach production areas globally. The objectives of this study were to (1) identify differentially expressed genes potentially involved in spinach downy mildew disease resistance based on *P. effusa*-spinach resistant reactions, and (2) examine gene expression of the obligate pathogen, *P. effusa*, during infection to identify potential race-specific virulence genes.

## Results

### Spinach host-P. effusa race compatibility, sample selection, and sequencing

The spinach cultivar Viroflay, and the near isogenic lines, NIL1, and NIL3, exhibited opposite resistant and susceptible reactions to the *P. effusa* races, R13 and R19 (Table 1). NIL1 was resistant to R13 while NIL3 was susceptible, and NIL3 was resistant to R19, while NIL1 was susceptible to R19. Viroflay was susceptible to both races. The results confirmed the variation in resistance across the selected NILs and the variation in virulence between the two *P. effusa* races as was intended for the objective of this study. For each of the treatments, R13-inoculated, R19-inoculated and mock-inoculated at each of the timepoints, 1 day post inoculation (dpi) and 6 dpi, three replicate samples were collected from Viroflay, NIL1, and NIL3 for a total of 54 samples. The 1 dpi timepoint represented early infection, before symptom development, while 6 dpi reflected later infection and *P. effusa* colonization as exhibited by light sporulation and leaf chlorosis (data not shown). Mock-inoculated plants did not exhibit symptoms.

**Table 1:** Compatibility of spinach cultivar or near isogenic lines (NILs) and *P. Effusa* races used in this study

	Treatment		
Cultivar	Mock	Race 13	Race 19
Viroflay	-	+	+
NIL1	-	-	+
NIL3	-	+	-

+ indicates a susceptible reaction to a given isolate or race

- indicates a resistant reaction to a given isolate or race

The 54 samples were subjected to RNA extraction, cDNA synthesis, library preparation, and sequencing. A total of 2,632,035,520 raw sequencing reads were generated from 53 samples in this study with one sample failing to get adequate sequencing reads which was one of the replicates for R19-inoculated NIL1 at 6 dpi (Supplementary File 1).

### Mapping of the transcriptome to the spinach reference genome

Of the raw sequencing reads for the 53 samples, an average of 35,027,245 reads (or 76.13%) mapped in pairs to the spinach genome (Supplementary File 1). An overall lower percentage of reads mapped to the spinach genome from the R19-inoculated susceptible cultivar Viroflay and the susceptible NIL1 at 6 dpi (ranging from 45.03 to 53.50%), indicating that an abundance of *P. effusa* reads were present in these samples. The percent reads mapped to spinach from the R13-inoculated susceptible cultivar Viroflay and the susceptible NIL3 was similar to the average, indicating that the abundance of *P. effsua* reads may have been lower relative to the R19-inoculated samples.

**Fig. 1:**
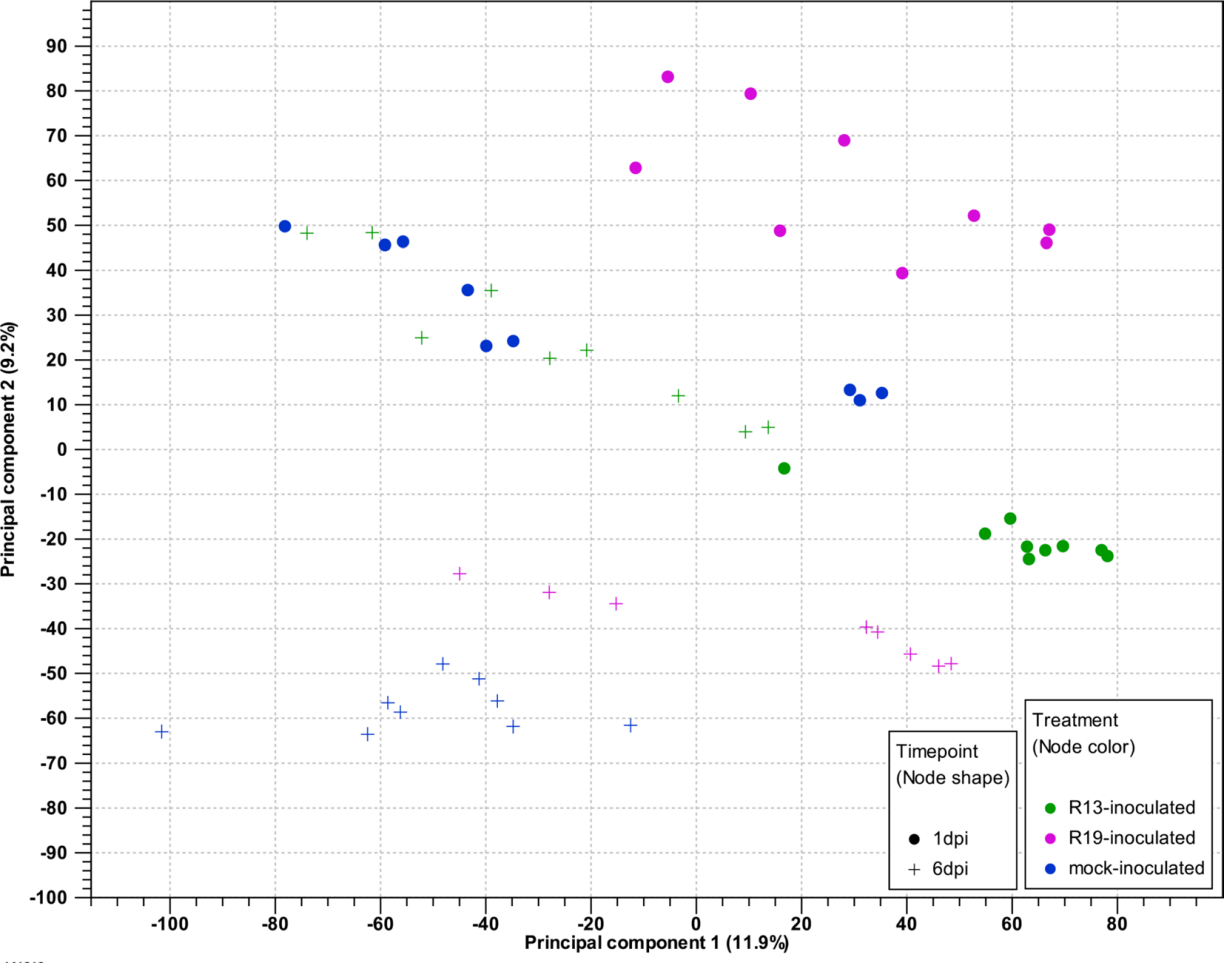
Principle component analysis (PCA) of total mapped reads from all *Peronospora effusa* and mock-inoculated samples mapped to the spinach genome. Samples include *P. effusa* R13-inoculated near isogenic spinach lines NIL1 and NIL3, and cultivar Viroflay plants at 1 day post-inoculation (dpi; green points) and 6 dpi (green crosses), *P. effusa* R19-inculated NIL1, NIL3, and Viroflay spinach plants at 1 dpi (pink points) and 6 dpi (pink crosses), and mock-inoculated NIL1, NIL3, and Viroflay spinach plants at 1 dpi (blue points) and 6 dpi (blue crosses)

To assess clustering of expression values across samples, principal component analysis (PCA) was performed using the reads mapped to spinach (Fig. 1). Clustering was observed for samples from the same treatment group and timepoint, indicating similarity in the expression profiles of the experimental replicates yet differences based on the experimental variables. R19-inoculated samples (pink) at both the 1 and 6 dpi timepoints clearly cluster from the mock-inoculated samples (blue), and R13-inoculated samples (green), cluster separately from R19-inoculated indicating differences in expression profiles in response to treatment with either *P. effusa* race. PC1 accounted for 11.9% of the variation in gene expression and PC2 accounted for 9.2%. PCA was also generated for spinach transcriptomes of R13 and R19-inoculated plants separately to highlight the spinach cultivar or near isogenic line expression profiles. These results indicated spinach plants that are resistant or susceptible to a given *P. effusa* race, cluster together, particularly at the later timepoint. For instance, clustering within the R19-inoculated samples was apparent for the resistant NIL3 at 6 dpi while the susceptible Viroflay and NIL1 clustered together at 6 dpi (Supplemental Fig. 1).

### Analysis of differentially expressed spinach genes in resistant reactions

**Fig. 2:**
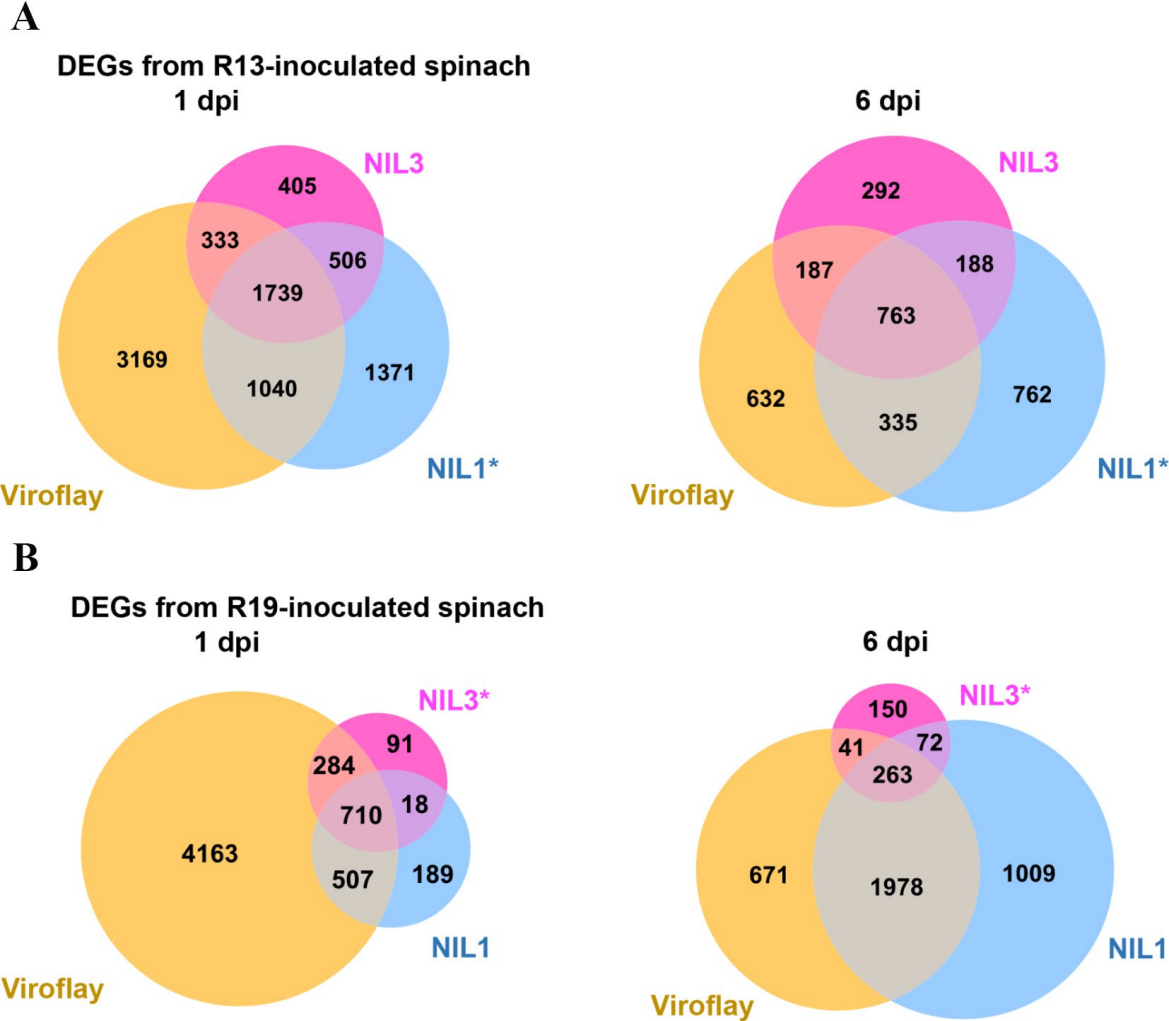
Differentially expressed genes (DEGs) across *Peronospora effusa* inoculated near isogenic spinach lines NIL1 and NIL3, and cultivar Viroflay. (**A**) Number of DEGs across *P. effusa* R13-inoculated spinach cultivars at 1 dpi (left) and 6 dpi (right). (**B**) Number of DEGs across *P. effusa* R19-inoculated spinach cultivars at 1 dpi (left) and 6 dpi (right). An asterisk (*) indicates the resistant cultivar to the corresponding *P. effsua* race or isolate with which plants were inoculated. DEGs have a log_2_fold-change cutoff of 1.5 and FDR p-value cutoff of 0.05

Pairwise comparisons of *P. effusa*-inoculated spinach transcriptomes to mock-inoculated transcriptomes were used to assess differentially expressed genes (DEGs). The total number of DEGs in R13-inoculated spinach was 8,563 at 1 dpi and 3,159 at 6 dpi and for R19-inoculated spinach, 5,962 total DEGs at 1 dpi and 4,184 at 6 dpi (Fig. 2, Supplementary File 2). Venn diagrams were used to highlight DEGs unique to a spinach host. R13-inoculated transcriptomes compared to mock-inoculated transcriptomes of the same spinach host identified 1,371 DEGs unique to the resistant reaction of R13-inoculated NIL1 at 1 dpi, and 762 DEGs at 6 dpi (Fig. 2A). For R19-inoculated transcriptomes compared to mock-inoculated, 91 DEGs were identified as unique to the resistant reaction of R19-inoculated NIL3 at 1 dpi, and 150 DEGs at 6 dpi (Fig. 2B).

**Fig. 3:**
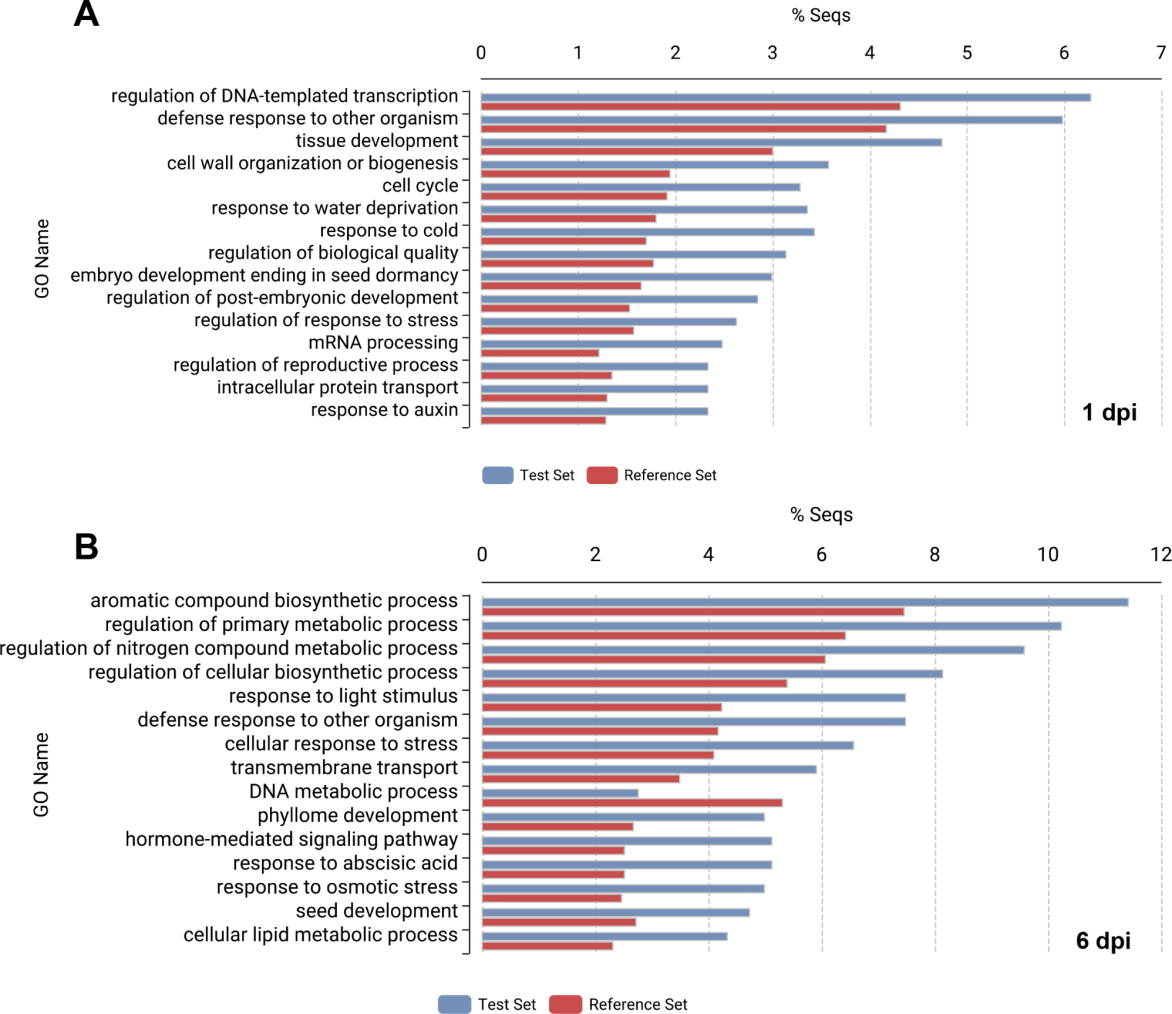
Enriched gene ontology (GO) categories from R13-inoculated NIL1 spinach plants at **A**) 1 dpi and **B**) 6 dpi. Enrichment is based on Fisher’s enrichment analysis of the 1,371 differential expressed genes (DEGs) from R13-inoculated NIL1 at 1 dpi and the 762 DEGs at 6 dpi which represent the Test Sets (in blue) compared to the 34,878 coding genes present in the Spinach v3 (Hulse-Kemp et al. 2021) as the Reference Set (in red).

**Fig. 4:**
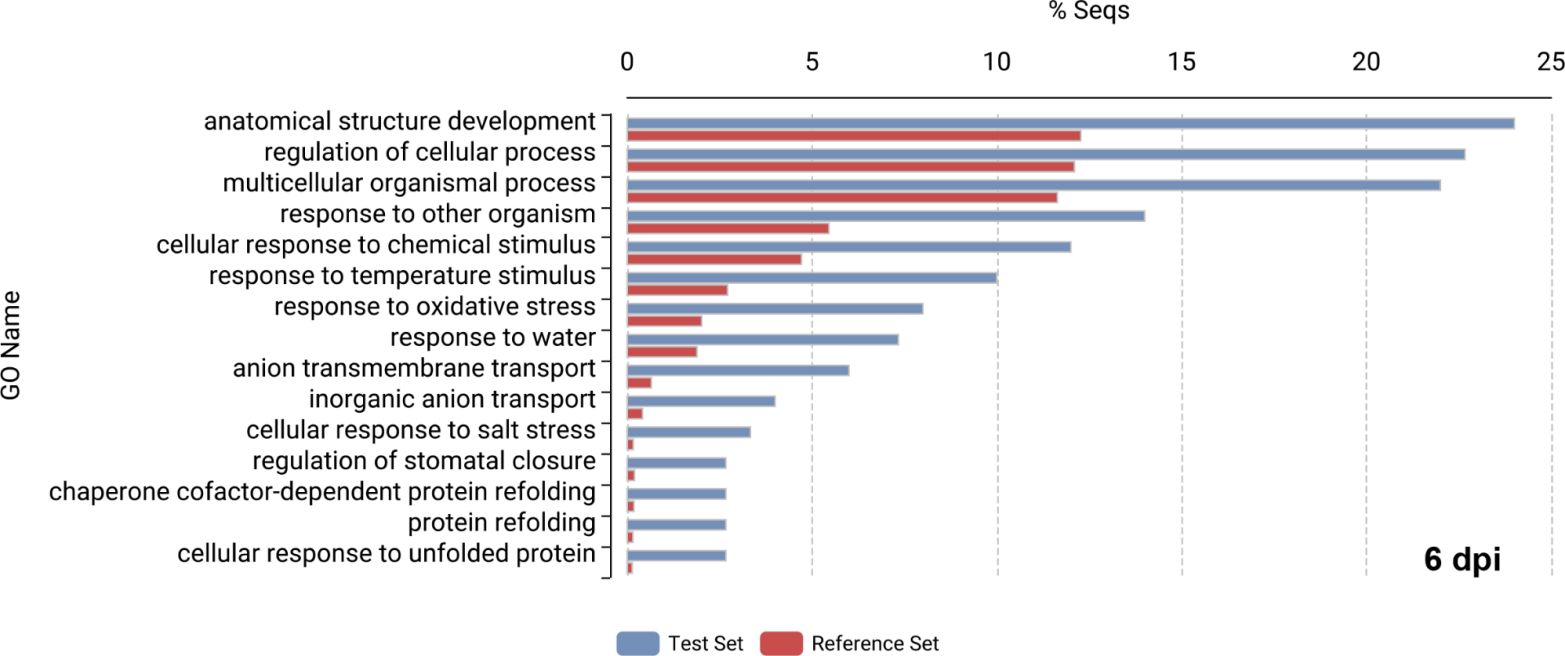
Enriched gene ontology (GO) categories from R19-inoculated NIL3 spinach plants at 6 dpi. Enrichment is based on Fisher’s enrichment analysis of the 150 differential expressed genes (DEGs) from R19-inoculated NIL3 at 6 dpi which represent the Test Sets (in blue), 34,878 coding genes present in the Spinach v3 (Hulse-Kemp et al. 2021) as the Reference Set (in red). Note that from the 91 DEGs present in R19-inoculated NIL3 at 1 dpi, no genes were considered enriched based on Fisher’s enrichment analysis.

To explore the spinach DEGs identified from the *P. effusa*-spinach resistant reactions, Fisher’s enrichment analysis was performed. The gene ontology (GO) category “defense response to other organism” or “response to other organism” was enriched in three of the resistant reaction DEG sets. For the 1,371 DEGs from R13-inoculated NIL1 spinach transcriptomes at 1 dpi roughly 6.0% of genes were enriched for “defense response to other organism” (Fig. 3A). Similarly, the 762 DEGs from R13-inoculated NIL1 transcriptomes at 6 dpi contained 7.5% of genes were enriched for this GO category (Fig. 3B). From the 150 DEGs from R19-inoculated NIL3 transcriptomes at 6 dpi, roughly 14.0% of DEGs were enriched for “response to other organism” (Fig. 4). The 91 DEGs from R19-inoculated NIL3 transcriptomes at 1 dpi did not contain any enriched DEGs based on Fisher’s enrichment analysis, which could have been due to the smaller sample size.

For the R13-inoculated NIL1 DEGs, several enriched genes from the “defense response to other organism” GO category, encode products of protein kinase-like and P-loop containing families (Tables 1 and 2 dpi, Tables 3 and 6 dpi). These included several receptor kinases such as cysteine-rich receptor-like protein kinases (CRKs), a putative receptor-like protein kinase which is a homolog of At4g00960, and a dual specificity protein kinase. A spinach homolog of brassinosteroid insensitive 1 (BAK1) was also upregulated at 1 dpi at a log_2_fold-change of 1.69. RGA-like disease resistance genes encoding P-loop motifs were upregulated in the enriched DEGs at 1 dpi with a log_2_fold-change of 3.31 and 11.1 and at 6 dpi with a log_2_fold-change of 4.62 and 8.93. At 1 dpi, two genes encoding cysteine protease-like proteins, the xylem-specific, XCP2-like, and the senescence-specific cysteine protease, SAG12-like, were also upregulated at 9.25 and 8.52 log_2_fold-change, respectively. However, at 6 dpi there were no cysteine protease-like encoded genes that were enriched.

**Table 2:** Enriched genes of interest from “defense response to other organism” GO category in R13-inoculated NIL1 spinach at 1 dpi

Gene ID	Description	Log_2_fold-change
**Protein kinase-like**		
Spiol01Chr13863	probable serine/threonine-protein kinase PBL7	2.44
Spiol01Chr14470	G-type lectin S-receptor-like serine/threonine-protein kinase At1g11300	7.83
Spiol0223C05961	BRASSINOSTEROID INSENSITIVE 1-associated receptor kinase 1-like (LRR)	1.69
Spiol0281C06495	putative receptor-like protein kinase At4g00960	9.89
Spiol02Chr28347	mitogen-activated protein kinase homolog MMK2-like	0.96
Spiol02Chr29971	phosphatidylinositol 4-kinase gamma 4-like	1.68
Spiol0309C00612	serine/threonine-protein kinase RIPK-like	-1.31
Spiol03Chr20502	dual specificity protein kinase shkC-like	1.01
Spiol04Chr09365	cold-responsive protein kinase 1-like isoform X2	3.59
Spiol04Chr09492	G-type lectin S-receptor-like serine/threonine-protein kinase At1g11300	2.28
Spiol04Chr10963	CBL-interacting protein kinase 32-like isoform X1	1.74
Spiol06Chr21559	cysteine-rich receptor-like protein kinase 10 (CRK)	-1.62
Spiol06Chr24027	cysteine-rich receptor-like protein kinase 10 (CRK)	8.02
***P-loop containing***		
Spiol04Chr08771	disease resistance protein RGA2-like (LRR)	3.31
Spiol04Chr10906	disease resistance protein RGA2-like	11.1
Spiol04Chr11086	AAA-ATPase At3g50940-like	-7.53
Spiol03Chr19080	dynamin-related protein 1E-like	7.83
Spiol03Chr20891	uridine-cytidine kinase C-like	3.04
Spiol06Chr21329	26 S proteasome regulatory subunit 8 homolog A-like	-0.85
Spiol06Chr21975	extra-large guanine nucleotide-binding protein 1-like	0.95
Spiol06Chr22567	probable RNA helicase SDE3	1.52
Spiol02Chr28975	ABC transporter G family member 39-like	8.68
***Cysteine protease-like***		
Spiol04Chr08923	cysteine protease XCP2-like	9.25
Spiol05Chr26712	senescence-specific cysteine protease SAG12-like	8.52

LRR = leucine-rich repeat domain

**Table 3:** Enriched genes of interest from “defense response to other organism” GO category in R13-inoculated NIL1 spinach at 6 dpi

Gene ID	Description	Log_2_fold-change
**Protein kinase-like**		
Spiol04Chr01972	leucine-rich repeat receptor protein kinase MSP1-like	5.03
Spiol04Chr08750	serine/threonine-protein kinase SAPK3-like	-2.92
Spiol04Chr09301	non-functional pseudokinase ZED1-like	-6.68
Spiol03Chr19209	probable serine/threonine-protein kinase PBL7	-9.06
Spiol03Chr20502	dual specificity protein kinase shkC-like	1.60
Spiol06Chr23414	serine/threonine receptor-like kinase NFP	2.19
Spiol05Chr26866	probable serine/threonine-protein kinase At4g35230	2.13
Spiol02Chr29971	phosphatidylinositol 4-kinase gamma 4-like	2.16
***P-loop containing***		
Spiol0290C06626	AAA-ATPase At3g50940-like	1.47
Spiol04Chr10024	endoribonuclease Dicer homolog 2-like	-9.26
Spiol01Chr13806	pre-mRNA-splicing factor ATP-dependent RNA helicase DEAH7-like	-9.10
Spiol01Chr14599	ras-related protein Rab2BV	-1.32
Spiol03Chr19889	disease resistance protein RPP8-like	2.97
Spiol06Chr22112	ABC transporter G family member 9-like	-4.17
Spiol05Chr27035	putative disease resistance protein RGA3	8.93
Spiol02Chr31491	putative disease resistance protein RGA3	4.62
Spiol0290C32209	AAA-ATPase At3g50940-like	4.87

The R19-inoculated NIL3 DEGs from 1 dpi and 6 dpi did not contain protein kinase-like and P-loop coding genes that were significantly enriched for the “response to other organism” GO category, however these gene families were still present in the DEG sets (Supplemental Tables 1 and Supplemental Table 2). This included the RGA3-like disease resistance protein, which was upregulated by 3.52 log_2_fold-change, and three receptor-like kinases, which were also upregulated by 1.47, 2.64, and 3.00 log_2_fold-change. Enriched DEGs from the R19-inoculated NIL3 resistance reaction at 6 dpi included genes encoding a proteinase inhibitor-like protein, an RD19A-like cysteine protease, a coronatine-insensitive 1 (COI1) protein, and several others (Supplemental Table 3).

### Distribution of DEGs across spinach chromosomes

**Fig. 5:**
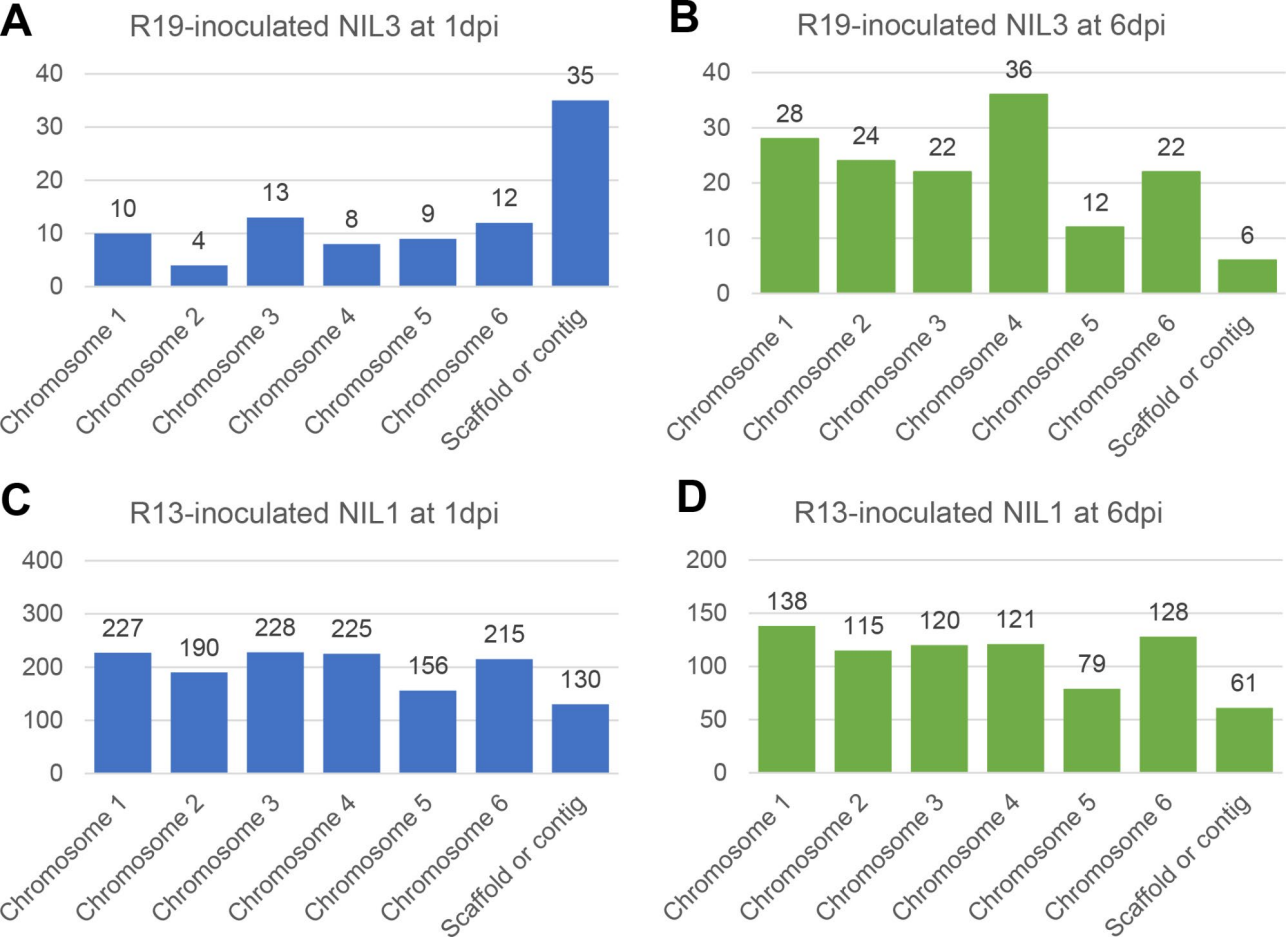
Distribution of differentially expressed genes (DEGs) in *Peronospora effusa*-spinach interactions by spinach chromosome. **A**) The 91 DEGs from R19-inoculated NIL3 at 1 dpi. **B**) The 150 DEGs at 6dpi. **C**) The 1,371 DEGs from R13-inoculated NIL1 at 1 dpi. **D**) The 762 DEGs at 6 dpi

Chromosome 3 of the spinach genome contains the RPF 1, 2, and 3 loci involved in downy mildew resistance [21, 25, 26]. To assess if the unique DEGs from the *P. effusa*-resistant cultivar reactions were in higher quantity on chromosome 3, the number of DEGs mapped across spinach chromosomes was compared. Interestingly, DEGs from the resistant reactions were not biased to any of the six spinach chromosomes (Fig. 5).

### Expression profile of resistance gene homologs in P. effusa-inoculated Viroflay, NIL1, and NIL3

In the spinach genome [27] used in this study, we identified eight homologs to the previously reported, potential resistance genes in spinach, Spo12784, Spo12903, and Spo12821 [25, 26] (Supplemental Table 4). All eight of the R gene homologs were annotated by InterPro Scan as NB-ARC domain-containing proteins and as part of the disease resistance protein family in plants. To analyze if gene expression varied for these eight homologs across the *P. effusa*-infected spinach hosts used in this study, their differential gene expression relative to mock-inoculated plants was compared. In the R13-inoculated NIL1 resistant reaction, the homologous resistance genes Spiol04Chr10906, Spiol01Chr02529, and Spiol03Chr02880 were upregulated at 1 dpi at 11.10, 8.07, and 2.10 log_2_fold-change, respectively (Supplemental Table 5). While in the R13-inoculated NIL3 susceptible reaction, two of these three genes were not expressed at the same timepoint. Similarly, in the R19-inoculated resistant NIL3, the homologous resistance genes Spiol04Chr11575 and Spiol01Chr02529 were upregulated 7.37 and 7.06 log_2_fold-change, respectively, at 1 dpi. In the R19-susceptible NIL1, only one of these two genes was upregulated, and the other was not expressed (Supplemental Table 6). In the universally susceptible cultivar, Viroflay, the R gene homologs were either not expressed, expressed at very low levels, or were downregulated. These results indicate differences in expression of these potential resistance genes across the near isogenic lines and the cultivar Viroflay when infested with *P. effusa* R13 vs. R19. Thus, these genes may be involved in race-specific host defense.

### Mapping of the transcriptome to the P. effusa reference genome

The unmapped reads from the initial mapping to the spinach genome were used to map to the *P. effusa* reference genome to establish the pathogen transcriptome in the different *P. effusa*-spinach interactions. The samples with the highest numbers of unmapped reads in spinach were the R19-inoculated NIL1 and Viroflay susceptible samples at 6 dpi, with a range of 39.20–48.72% unmapped reads (Supplemental File 1). Of these samples, most of the reads unmapped to spinach were mapped to the *P. effusa* genome in pairs at a range of 82.61–89.46%, indicating an abundance of pathogen reads and read coverage. The R13-inoculated samples had an overall lower percentage of reads unmapped to the spinach genome from the R13-inoculated NIL3 and Viroflay susceptible samples at a range of 5.19–8.06%. Of these samples, 11.03–13.51% mapped to the *P. effusa* genome (Supplemental File 1).

**Fig. 6:**
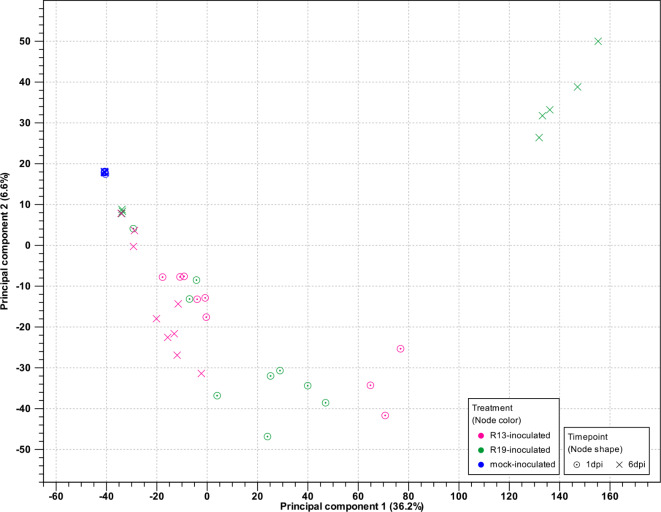
Principle component analysis (PCA) of reads unmapped to the spinach reference genome that mapped to the *Peronospora effusa* genome. *P. effusa* R13-inoculated samples (pink), *P. effusa* R19-inculated samples (green) and mock-inoculated samples (blue) with 1 dpi (open circles) and 6 dpi (crosses)

From the reads that mapped to *P. effusa*, clustering by PCA was observed for samples from the same treatment group and timepoint (Fig. 6). *P. effusa* mapped reads from the mock-inoculated samples clearly clustered together and were separate from the pathogen-containing samples, these reads likely contain contaminants as *P. effusa* was not present in these samples. The R19-inoculated susceptible samples at 6 dpi had the highest percentage of reads mapped to *P. effusa* and clustered separately from R13 at 1 and 6 dpi and R19 at 1 dpi, indicating differences in the transcripts between the two races and timepoints.

### Analysis of race-specific DEGs in P. Effusa

To identify potentially race-specific DEGs associated with virulence in *P. effusa*, pairwise comparisons were performed on the transcriptomes of the *P. effusa*-inoculated samples, comparing 6 dpi and 1 dpi to identify early and late DEGs following inoculation. These comparisons included R19 on NIL1 at 6 dpi vs. 1 dpi, R13 on NIL1 at 6 dpi vs. 1 dpi, R19 on NIL3 at 6 dpi vs. 1 dpi, R13 on NIL3 at 6 dpi vs. 1 dpi, R19 on Virolfay at 6 dpi vs. 1 dpi, and R13 on Virolfay at 6 dpi vs. 1 dpi (Supplementary File 3). To ascertain which of these DEGs were expressed by a given race, Venn diagrams were generated to compare DEGs from R13 to R19 on the differentiating NILs and Viroflay (Supplemental Fig. 2). 201 DEGs were identified from R19 as opposed to R13 during infection of NIL1 and 732 DEGs were identified from R13 as opposed to R19 during infection of NIL3. Additionally, on the universally susceptible host, Viroflay, there were 1307 DEGs identified from R19, 381 DEGs identified from R13, and 610 DEGs expressed by both R13 and R19.

Of the DEGs, several were of interest as potential virulence genes. From R19, three RxLR-type and one crinkler-type effector were downregulated based on the pairwise comparison, indicating their expression at the 1 dpi timepoint and their potential role in pathogen establishment within the host (Table 4). R13 also expressed several effector proteins during infection of NIL3 at both 1 and 6 dpi (Table 5). In addition, four necrosis inducing-like proteins were upregulated, indicating that their increased expression at 6 dpi had occurred later in the infection cycle. DEGs including two of the necrosis inducing-like proteins, two of the predicted RxLRs, two of the sugar transport proteins and several proteases or protease inhibitors were also differentially expressed in either R13 or R19 when inoculated on Viroflay indicating their conserved expression during *P. effusa* infection while other DEGs remain specific to R13 or R19 on NIL3 or NIL1, respectively (Tables 4 and 5; Supplementary File 4). Several hypothetical proteins were also identified as DEGs with 83 hypothetical proteins from the R13 on NIL3 and 27 from the R19 on NIL1 elucidating other potential virulence genes with currently unknown functions.

**Table 4:** *Peronospora effusa* virulence gene homologs from *P. effusa* R19 on NIL1

Name	Description		Log_2_fold-change^A^
**Early DEGs - expressed at 1 dpi**			
Peff_UA202013_009717		Protein kinase-like domain	-6.65
Peff_UA202013_007568		RXLR-class effector Avh205	-5.57
Peff_UA202013_009600		Crinkler (CRN) family protein	-5.47
Peff_UA202013_000334		RXLR effector Avh1	-5.12
Peff_UA202013_004961^B^		elicitin-like protein INL3B	-4.49
Peff_UA202013_004673		Necrosis inducing-like protein NPP1 type	-3.88
Peff_UA202013_001098^B^		Cysteine-rich secretory protein family	-3.54
Peff_UA202013_002532^B^		Secreted RxLR effector peptide protein	-2.77
***Late DEGs - expressed at 6 dpi***			
Peff_UA202013_005449^B^		Glycoside hydrolase superfamily	3.57
Peff_UA202013_004791^B^		Sugar transport protein 10	3.89
Peff_UA202013_008922^C^		Secretory protein OPEL	4.68
Peff_UA202013_007937		Elicitin	4.82
Peff_UA202013_009055^C^		Dual specificity protein phosphatase 1	5.26

^A^Negative log_2_fold-change values represent expression at 1 dpi and positive log_2_fold-change values representexpression at 6 dpi due to the pairwise comparison of 6 dpi to 1 dpi for the R19-inoculated samples

^B^Differentially expressed in *P. effusa* R19 when inoculated on Viroflay

^C^Differentially expressed in *P. effusa* R13 when inoculated on NIL3

**Table 5:** *Peronospora effusa* virulence gene homologs from *P. effusa* R13 on NIL3

Name	Description	Log₂ fold-change
**Early DEGs - expressed at 1 dpi**		
Peff_UA202013_003042^B^	Protease inhibitor protein	-11.32
Peff_UA202013_002276^B^	Papain-like cysteine protease C1	-9.65
Peff_UA202013_005815^B^	RxLR-like protein	-8.80
Peff_UA202013_008116	Avr1b-1 Avirulence-like protein	-8.60
Peff_UA202013_008560	RxLR-like protein	-7.11
Peff_UA202013_006272	RxLR effector protein	-6.89
Peff_UA202013_007762^B^	Avirulence protein (Avh)	-6.67
Peff_UA202013_006206	Avirulence (Avh) protein	-6.66
Peff_UA202013_002775	RxLR-like protein	-6.60
Peff_UA202013_002787	Secreted RxLR effector peptide protein	-6.59
Peff_UA202013_008920	Cysteine protease ATG4B	-6.48
Peff_UA202013_004853^B^	Sugar (and other) transporter	-6.47
Peff_UA202013_008639^B^	Calpain-like cysteine protease C2	-6.42
Peff_UA202013_006259	Avirulence protein (Avh)	-6.33
Peff_UA202013_002794	Secreted RxLR effector peptide protein	-6.31
Peff_UA202013_001060^B^	Protease inhibitor EpiC4	-2.86
***Late DEGs - expressed at 6 dpi***		
Peff_UA202013_005599	NPP1 protein	2.84
Peff_UA202013_008922^C^	Secretory protein OPEL	3.60
Peff_UA202013_004663^B^	necrosis inducing-like protein NPP1 type	4.49
Peff_UA202013_004661^B^	necrosis inducing-like protein NPP1 type	4.67
Peff_UA202013_002867	Putative RxLR effector	4.82
Peff_UA202013_002408^B^	Glycoside hydrolase catalytic N-terminal domain-containing protein	6.56
Peff_UA202013_008429	secreted RxLR effector peptide protein, putative	6.77
Peff_UA202013_009055^C^	Dual specificity protein phosphatase 1	6.80
Peff_UA202013_009439	secreted RxLR effector peptide protein, putative	6.98
Peff_UA202013_004668	necrosis inducing-like protein NPP1 type	7.45
Peff_UA202013_002407	Glycoside hydrolase catalytic N-terminal domain-containing protein	9.34

^A^Negative log_2_fold-change values represent expression at 1 dpi and positive log_2_fold-change values represent expression at 6 dpi due to the pairwise comparison of 6 dpi to 1 dpi for the R13-inoculated samples

^B^Differentially expressed in *P. effusa* R19 or R13 when inoculated on Viroflay

^C^Differentially expressed in *P. effusa* R19 when inoculated on NIL1

## Discussion

Here we present and summarize the transcriptomes of the spinach cultivar Viroflay and the near isogenic lines NIL1 and NIL3 in response to infection with *P. effusa* race 13 or 19, in addition to transcriptomic analysis on the two pathogen races during infection. By utilizing NILs with differing compatibility to the *P. effusa* races used for infection, we were able to focus on the transcripts expressed in resistant spinach reactions.

Of the spinach DEGs identified from the resistant reactions, including those enriched for the GO category “defense response to other organism”, many encoded protein kinase-like or P-loop containing proteins. Protein kinases are involved in multiple cellular processes, particularly in transmitting responses to environmental, abiotic, and biotic signals [35]. Thus, there is a large selection of different protein kinases. Of them, receptor-like kinases (RLKs) have been identified as key players in both plant development and defense [36]. RLKs typically contain an extracellular domain for signal perception, a transmembrane domain, and an intercellular kinase domain for signal transduction. In host defense, RLKs directly or indirectly perceive pathogen associated molecular patterns (PAMPs) triggering a downstream immune response [37, 38]. Of the DEGs from the resistant *P. effusa*-spinach reactions, Spiol0281C06495 was annotated as a putative receptor-like protein kinase with homology to the Arabidopsis RLK At4g00960 in the R13-inoculated NIL1 resistant reaction at 1 dpi. In the same resistance reaction two cysteine-rich receptor-like protein kinase 10 (CRKs), Spiol06Chr21559 and Spiol06Chr24027 were also differentially expressed. CRKs are a subfamily of RLKs that contain two copies of a DUF26 domain which is made up of conserved cysteine residues, they are known to regulate several biological processes including disease resistance and cell death [39]. Expression of CRK28 in Arabidopsis increased host resistance to the bacterial pathogen, *Psuedomonas syringae* [40]. The same study also found CRK28 to associate with brassinosteroid insensitive 1-associated receptor kinase (BAK1) as part of the flagellin-sensing 2 (FLS2)-BAK1 immune complex. Interestingly, a BAK1 homolog, Spiol0223C05961, was found to be enriched and upregulated in the R13-inoculated resistant NIL1 transcriptome at 1 dpi as well. BAK1 is a well-known co-receptor of FLS2 of which activation leads to PAMP-triggered immunity (PTI) in the host [41, 42]. Thus, identification of novel RLKs that interact with BAK1 and its homologs could also lead to identification of RLKs that recognize PAMPs.

Several DEGs from both the R13-NIL1 and R19-NIL3 resistant reaction transcriptomes were probable leucine-rich repeat (LRR) receptor-like protein kinases or contained an LRR motif including Spiol04Chr08771, Spiol04Chr01972, Spiol05Chr26812, Spiol04Chr11049, Spiol0084S08137, Spiol03Chr20299, and the BAK1 homolog, Spiol0223C05961. LRR-RKs are the largest subfamily of transmembrane bound RLKs in plants and LRRs are known to play a significant role in plant defense [43, 44]. BAK1, for example, contains an LRR motif which is responsible for signal recognition [42]. FLS2 is another well-known LRR-RLK which recognizes flagellin protein, flg22 [37, 41]. More specifically to downy mildews, the *Bremia lactucae* resistance gene from lettuce, *Dm3*, encodes a nucleotide-binding site (NBS)-LRR [45]. The spinach transcriptome study by Kandel et al. 2020 also identified an LRR-RLK (Spiol05Chr34466) and a serine/threonine RLK1 (Spiol04Chr12186) to be upregulated in the spinach cultivar Solomon at 48 hpi with *P. effusa* but downregulated at later timepoint [34]. Thus, the spinach LRR protein-kinase like and other RLKs identified in the resistant host transcriptomes are likely involved in *P. effusa* pathogen recognition or other immune signaling events during defense.

Several P-loop containing proteins were also present in the DEGs from the *P. effusa*-spinach resistant reactions. The P-loop motif is a phosphate binding loop found in NTPase enzymes which mediates binding and transfer of NTP terminal phosphate groups [46, 47]. Structural models for NBS domains often contain P-loops which assist in interaction with ADP [48, 49]. Several P-loop containing, resistance gene analogs (RGA)-like were identified from our resistant spinach transcriptomes including the RGA2-like, Spiol04Chr08771 and Spiol04Chr10906 which were both upregulated in R13-inoculated NIL1 at 1 dpi. RGA2 has previously been shown to confer resistance to *Phytophthora infestans* in potato and to be expressed in a *P. capsici*-resistant pepper cultivar upon infection [50, 51]. Additionally, the RGA3-like disease resistance proteins Spiol05Chr27035 and Spiol02Chr31491 were upregulated in R13-NIL1 at 6 dpi and Spiol0329C00667 was upregulated in the R19-NIL3 resistant reaction at 1 dpi.

ABC transporters which are part of the ATP-Binding Cassette superfamily were also present in the P-loop containing DEGs from the resistant spinach reactions. Including Spiol02Chr28975 in the R13-NIL1 transcriptome at 1 dpi, Spiol06Chr22112 at 6 dpi, and Spiol06Chr03998 and Spiol04Chr09723 in the R19-NIL3 transcriptome at 6 dpi. Plant ABC transporters are involved in many functions including response to stress and pathogen resistance [52]. For example, an ATP-dependent binding cassette transporter G family member was previously shown to aid in abscisic acid tolerance, contributing to basal resistance against *P. syringae* in Arabidopsis [53]. Interestingly, the ABC transporter identified from our analysis at 1 dpi was highly upregulated while the transporters at 6 dpi were downregulated indicating their potential functions may be more relevant during the initial host response to *P. effusa*.

Cysteine protease-like DEGs were also found with two being highly upregulated in the R13-NIL1 1 dpi transcriptome and one being downregulated in the R19-NIL3 6 dpi transcriptome. All three cysteine proteases identified in our resistant reaction transcriptomes belong to the papain-like cysteine protease (PLCP) family [54]. PLCPs are key players in plant host defense and are known to be targeted by effectors of bacterial, fungal, oomycete, and nematode pathogens [55]. Upregulation in PLCP transcript and protein abundance has also been observed in citrus in response to the bacterial pathogen *Candidatu*s Liberibacter asiaticus (*C*Las) in addition to inhibition of PLCP activity by a *C*Las effector [56]. Similarly in tomato, the PLCP C14 is targeted by the *Phytophthora infestans* effectors EPIC1 and EPIC2B along with the PLCPs PIP1 and RCR3 which are targeted by the *Cladosporium fulvum* effector, Avr2 [57, 58]. Therefore, PLCPs may also play a role in spinach defense against *P. effusa*, specifically during the early stage of R13 infection of NIL1.

We also compared differential gene expression of eight homologs to previously identified R genes in spinach. Notably, Spiol04Chr10906, which is a P-loop containing RGA2-like protein was one of the R gene homologs, with homology to Spo12784 [25]. Spiol04Chr10906 is differentially expressed in NIL1 post inoculation with R13 relative to mock, but not in NIL3 indicating a potential role for this protein in host-specific defense. It should be mentioned that Spiol04Chr10906 is also expressed in NIL1 when inoculated with R19, therefore, it may only play a role in defense against multiple *P. effusa* races. Of the eight R gene homologs, expression in Viroflay was either non-existent, downregulated, or at a lower log_2_fold-change compared to expression of these genes in the NILs, potentially signifying that the universal susceptibility of Viroflay could be because these genes are not expressed in as high abundance. Surprisingly, the homologs were not all located on Chromosome 3, where the predicted R genes originated as part of RPF 1 or 2, thus, spinach resistance genes are likely distributed across the genome, which was also noted by the distribution of DEGs in the *P. effusa*-spinach disease resistant reactions across the spinach chromosomes.

In this study we utilized the near isogenic lines with differing compatibility to *P. effusa* R13 and R19 to investigate gene expression in resistant interactions; however, further studies using commercial spinach cultivars with claimed, race-specific resistance would provide more information on field-applicable resistance. For instance, Yakalo and Caladonia are resistant to both R13 and R19, yet, Meerkat and Hydrus are resistant to R13, while they are susceptible to R19.

Obligate oomycete pathogens, such as the downy mildews, rely on their hosts for survival, therefore, effectors and other virulence factors which facilitate colonization and modulate host metabolism to their benefit are essential [59, 60, 61, 62]. By mapping the unmapped reads from spinach to the *P. effusa* genome, we were able to assess potential virulence genes from *P. effusa* R13 and R19 during infection of the susceptible hosts NIL3 or NIL1, respectively, and the universally susceptible Viroflay. We chose to analyze differential gene expression of the pathogen on susceptible hosts due to the higher pathogen presence in the samples and therefore increased percentage of reads mapped to *P. effusa*. Early DEGs, expressed at 1 dpi by R19 included three RxLR effectors and one crinkling and necrosis (CRN) family protein. Effectors with an N-terminal RxLR motif (Arginine-any amino acid-Leucine-Arginine) are well documented virulence proteins of oomycetes and multiple examples of RxLRs in suppression of host immunity have been published [59, 63–65]. These include the *Phytophthora infestans* effector, AVRblb2, which prevents host secretion of a defense-related protease and the *Plasmopara viticola*, PvRXLR111 effector which increased host susceptibility by potentially stabilizing the transcription factor, WRKY40 [64, 65]. In another study, several other *P. viticola* RxLR effector candidates suppressed programmed cell death initiated by various elicitors when infiltrated in *N. benthamiana* [66]. CRNs are also cytoplasmic effectors which are widespread across oomycetes and although they have been less extensively researched, they are thought to have an equal role in pathogen virulence [67]. The *P. capsici* CRN, CRN12_997 binds to a host transcription factor and inhibits immunity in tomato [68]. One CRN and three RxLR effectors identified from sunflower downy mildew, *Plasmopara halstedii*, were shown to induce a hypersensitive-like cell death in some sunflower NILs [69]. Thus, the CRN and the RxLR effectors identified here from *P. effusa* growing on susceptible NILs likely play a role in evading host immunity, allowing for successful colonization and infection.

CRNs of *P. capsici* have been shown to be upregulated at both early and late stages of infection [70] while RxLRs are thought to be associated with the early or biotrophic stage of infection [71]. The previous transcriptomic study on *P. effusa*-spinach interactions found CRN-like effectors to be expressed later during infection, while RxLRs were expressed earlier [34]. Here we only identified the one CRN, Peff_UA202013_009600, expressed at 1 dpi, from R19 on its susceptible host NIL1. RxLRs were expressed by R19 on the susceptible NIL1 at the early infection stage, however, by R13 on the susceptible NIL3, RxLRs were expressed at both the early and late stages of infection. Considering obligate downy mildews are biotrophic pathogens, expression of effectors to suppress host immunity likely occurs in multiple waves through the pathogen’s lifespan [72]. Notably, the predicted RxLRs, Peff_UA202013_005815 and Peff_UA202013_002794 were also expressed by R13 and R19 on Viroflay, while differential expression of RxLRs, Peff_UA202013_008560, Peff_UA202013_006272, Peff_UA202013_002787, Peff_UA202013_002867, Peff_UA202013_008429, and Peff_UA202013_009439 were unique to R13 on NIL3. Similarly, the predicted RxLR, Peff_UA202013_002532, was expressed by R19 on both susceptible hosts NIL1 and Viroflay, while Peff_UA202013_007568 and Peff_UA202013_00033 where only expressed by R19 on NIL1 indicating potential host specificity of certain RxLRs.

Additionally, we found several necrosis inducing-like proteins expressed at the later infection stage of R13 on NIL3, R13 on Viroflay, and one expressed by R19 on NIL1 at 1 dpi. Necrosis-inducing *Phytophthora* protein 1 (NPP1) has been shown to induce pathogenesis-related (PR) gene abundance, ROS production, and an HR-like cell death in Arabidopsis and parsley, thus serving as an elicitor of host defense [73]. There are examples of necrosis inducing-like proteins contributing to pathogen virulence, but this is mainly during the necrotrophic phase of hemibiotrophic pathogens [74]. Kandel et al. 2020 also identified an NPP1 type necrosis inducing-like protein from *P. effusa* at the late stage of infection on the spinach cultivar Viroflay [34]. The repeated appearance of NPP1 type proteins in the *P. effusa* transcriptome during infection indicates they have some role in pathogenesis and their elicitor function is possibly masked by other virulence proteins. Interestingly, the secretory protein OPEL was expressed at the later infection timepoint for both R13 and R19 on NIL3 and NIL1, respectively, and not Viroflay. OPEL is an apoplastic elicitor of *Phytophthora parasitica*, which has homologs only in oomycetes, and induces cell death, callose, ROS and SA-responsive defense genes in tobacco [75]. Considering both R13 and R19 can establish infection on NIL3 and NIL1, respectively, recognition of the *P. effusa* OPEL elicitor might also be subverted by an effector or potentially not recognized in these hosts. An effective *P. effusa* elicitor could be a useful tool in assaying effector function and screening resistant spinach cultivars.

Importantly, at 1 dpi, pathogen abundance was less, as indicated by the lower percentage of mapped reads to the *P. effusa* genome, compared to 6 dpi, and the variation of early timepoints in the PCA analysis. Thus, additional timepoints could provide a more robust illustration of early virulence gene expression. Another inspiration for future studies is the *P. effusa*-associated microbiome and potential contaminants that could be present in the remaining unmapped reads as an entire metagenome is present when sequencing obligate pathogens [76].

## Conclusions

Here we present a dual transcriptome analysis of *P. effusa* and spinach using different pathogen races and spinach NILs as tools to compare race- and host-associated genes expressed during downy mildew pathogenesis. The transcriptomic data for both spinach and *P. effusa* during compatible and incompatible interactions are valuable resources for continuing exploration of *P. effusa* virulence genes and spinach defense or resistance genes. Future functional analysis of the genes discussed will help to shed light on their roles in downy mildew disease and facilitate breeding efforts.

## Methods

### Plant and pathogen materials

Spinach plants of cultivar Viroflay and the near-isogenic lines, NIL1 and NIL3, were grown from seed (provided by Naktuinbouw, the Netherlands) in trays filled with potting mix (Sunshine LC1, Sungro Horticulture, Canada). Four trays with approximately 100 spinach plants were grown for each cultivar. Plants were grown in growth chambers at a constant temperature of 19ºC with a 12 h light/dark cycle. Two-week-old seedlings were used for inoculation.

Plants were inoculated with *P. effusa* isolates UA202001E (Race 19) or UA0510C (Race 13). Sporangia suspensions of each isolate were prepared by scraping sporangia from spinach leaves into cold water followed by filtering the sporangia solution through cheesecloth. A 20 ml suspension with a concentration of 10^5^ sporangia/ml was used to spray on each tray using a Badger Basic Spray Gun (Model 250). The suspension was also sprayed onto water agar plates and kept in a dew chamber for 24 h to validate sporangia germination.

Inoculated plants were kept in a dew chamber with no light for 24 h at 19 °C to maintain free moisture, and subsequently transferred to isolated growth chambers under a cycle of 12 h of light and 12 h of darkness and a constant temperature of 19ºC. At 6 days post-inoculation (dpi), plants were moved back to the dew chamber and incubated for an additional 24 h to induce sporulation. Disease symptoms, including leaf chlorosis and light sporulation were observed on susceptible plants 5 dpi. Mock-inoculated plants were treated with water. Mock-inoculated controls were kept in separate dew chambers under the same conditions and did not exhibit downy mildew disease symptoms.

### Sample collection

Samples were collected at 1 day post-inoculation (dpi) and at 6 dpi. Each sample consisted of the leaves of three plants which were pooled following treatment with either R13 or R19 of *P. effusa*. Samples were immediately frozen in liquid nitrogen and kept at -80ºC until RNA extraction. At each time point, three replicate samples were collected from cultivar Viroflay, NIL1, and NIL3 for a total of 54 samples. These samples were used for RNA extraction.

### RNA extraction, library preparation, and sequencing

For RNA extraction, 100 mg of frozen leaf tissue was weighed and transferred to a sterile 1.5 ml tube. The RNeasy Plant Mini Kit (Qiagen, Valencia, CA) was used for RNA extraction. Briefly, samples were pulverized in with a sterile metal bead in 450 µl of lysis buffer containing 10 µl of β-mercaptoethanol using a pre-chilled block on a Tissulyser (Qiagen). The manufacturer’s protocol was followed except at the RWI step, which was performed in two subsequent aliquots of 350 µl, as opposed to one of 700 µl. The RNA was quantified and checked for quality using a Nanodrop 8000 (Thermo Fisher Scientific, Waltman, MA).

Turbo DNase (Invitrogen, Waltman, MA) was added to remove residual DNA from the RNA extractions in a ratio of 1 µl of DNase per 1 µg of RNA. Post DNase treatment RNA was quantified again using a Qubit Fluorimeter and the Broad Range Qubit RNA assay kit (Invitrogen).

Synthesis of cDNA, library preparation, and sequencing were performed by Novogene (Sacramento, CA). The mRNA libraries were prepared by poly A enrichment using Oligo-dT beads. Libraries were sequenced on a NovaSeq 6000 Illumina (San Diego, CA) platform using 150 bp pair-end reads. 54 samples were submitted but sequencing failed for one sample. A total of 2,632,035,520 raw reads for the 53 samples were obtained and are deposited as BioProject ID: PRJNA1104270 in NCBI. The average Phred quality score (Q30) was 94.9%.

### Read trimming and mapping to spinach and P. effusa genomes

Raw reads from the 53 sequenced samples were imported as pairs to CLC Genomics Workbench v21.0.5 (Qiagen). Adapter sequences and low-quality reads were removed using “Trim reads” function with the default settings in addition to removing reads less than 100 bp in length. The average number of clean reads per sample was 46,017,450 (Supplementary File 1). The trimmed and cleaned reads were mapped to the Spinach v3 genome [27] using the “RNA-Seq Analysis” tool with the “genome annotated with genes and transcripts” option to generate expression tracks. Reads were either mapped in pairs, or in broken pairs, reads mapped in broken pairs were considered as single reads and were not counted in subsequent analysis. Only reads that mapped as pairs were used to increase confidence in transcript predictions. Mapped fragments (FPKM) were counted as one when two reads mapped as an intact pair. An average of 35,027,245 reads (or 76.13%) mapped in pairs to the spinach genome (Supplementary File 1). R19-inoculated samples at the 6 dpi timepoint had overall lower percentage of reads that mapped to spinach, (range from 45.03 to 59.47%) indicating more *P. effusa* material was present.

Following the spinach mapping, unmapped reads were used to map to the *P. effusa* genome, isolate UA202013 [31]. Reads that mapped to *P. effusa* in broken pairs were not used in subsequent analysis. Only reads from the R19-inoculated, 6 dpi timepoint samples mapped with higher than 50% of reads to the *P. effusa* genome, with an average mapping of 87.17%.

*Reference genomes*.

The reference spinach genome used in this study was the cultivar Viroflay, which was sequenced using Pacific Biosciences long-read technology to a total of ~ 70X coverage and anchored to individually assembled Illumina short-reads to generate a chromosome level spinach genome [27]. The reference *P. effusa* genome [31] was from isolate UA202013 and does not correspond to a currently named race. The genome was sequenced using Pacific Bioscience high-fidelity reads to a ~ 31X whole-genome coverage and assembled to generate 17 telomere-to-telomere chromosomes [31].

### PCAs, differential gene expression, and gene ontology (GO) enrichment analysis

Principle component analysis (PCA) and pairwise comparisons for differential gene analysis were performed in CLC Genomics Workbench v21.0.5. Log counts per million (CPM) were calculated for each gene based on the TMM-adjusted (trimmed mean of M values) values [77], followed by a Z-score normalization across samples. PCA plots were generated with the “PCA for RNA-Seq” tool using the normalized logCPM from the mapped reads to spinach and to *P. effusa* for all 53 samples as expression values. Pairwise comparisons of different samples were performed to determine differentially expressed genes (DEGs) using the “Differential Expression for RNA-Seq” tool which applied the GLM (generalized linear model) multi-factorial statistical formalism and the Wald test across comparisons [78]. For spinach, comparisons were between *P. effusa*-inoculated and mock-inoculated samples, using the mock-inoculated samples as the control groups. For *P. effusa*, pair-wise comparisons were between the inoculated samples of either R19 or R13 at 6 dpi and 1 dpi, using 1 dpi as the control group. The comparisons were used to generate Venn Diagrams and DEGs displayed in the Venn Diagrams were filtered to a log_2_fold-change of ± 2.0 and FDR p-value of *≤* 0.05 for spinach and a p-value *≤* 0.05 for *P. effusa*.

Functional analysis and gene ontology (GO) enrichment were performed in OmicsBox v3.0.29 (BioBam, Valencia, Spain) with the Functional Analysis module. DEG sequences were imported and Blast to a query database using the dedicated CloudBlast computing cloud. For spinach, sequences were blast-x against a non-redundant protein seq (nr v5) database with a taxonomy filter for Chenopodiaceae and Amaranthaceae, and for *P. effusa* a taxonomy filter of Peronosporales and Oomycota. For spinach, blast analysis was followed by InterProScan annotation [79, 80] and InterProScan GO merging with Blast results, GO mapping and GO annotation [81]. Enrichment analysis was performed on the sets of DEGs found in resistant spinach reactions using Fisher’s Exact Test by comparing the DEGs in those groups to all spinach genes [82].

For each of the resistant spinach reactions, DEG distribution across chromosomes was quantified for chromosomes 1–6 and the remaining scaffolds or contigs. The number of DEGs per chromosome was compared to assess if chromosome 3 had more DEGs.

### Differential gene expression analysis of potential resistance genes across spinach cultivars

The coding sequences of the potential downy mildew resistance genes Spo12784, Spo12903 and Spo12729 from She et al. 2018 [25] and Spo12821 from Gao et al. 2022 [26] were extracted from the SpinachBase (spinachbase.org) genome portal [83] and used in blast searches against the Spinach v3 CDS [27] to find homologs. A gene was considered a homolog if more than 60% identify and 40% overlap were met. No homologs met these criteria for Spo12729 so it was removed from the analysis. Some genes were homologs of more than one of the three previously predicted potential R genes (Supplemental Table 4). The eight homologs identified were examined for differential gene expression across the *P. effusa*-infected spinach cultivar, Viroflay and the NILs. Log_2_fold-change values with p-values of 0.05 or less were extracted for the homologous genes from the pairwise comparisons (*P. effusa*-inoculated vs. mock-inoculated) for each *P. effusa* race on each spinach type at each timepoint.

## Electronic supplementary material

Below is the link to the electronic supplementary material.

Supplementary Material 1

Supplementary Material 2

Supplementary Material 3

Supplementary Material 4

Supplementary Material 5

## Data Availability

Sequence data that support the findings have been deposited in the National Center for Biotechnology Information as BioProject PRJNA1104270.
