# Supplementary material for: Dual transcriptional characterization of spinach and Peronospora effusa during resistant and susceptible race-cultivar interactions
Source: BMC Genomics. 2024 Oct 7;25:937. doi: 10.1186/s12864-024-10809-x (PMC11457348; doi:10.1186/s12864-024-10809-x)
Supplement: Supplementary file 5 — Supplementary Material 5 [file 12864_2024_10809_MOESM5_ESM.docx]

Dual transcriptional characterization of spinach and *Peronospora effusa* during resistant and susceptible race-cultivar interactions.

Kelley J. Clark, Amy G. Anchieta, Chunda Feng, Allen Van Deynze, James C. Correll, and Steven J. Klosterman

Supplementary information

Additional file 2: Contains supplemental figures and tables.

Supplemental Fig. 1. Principle component analysis (PCA) of total mapped reads of *Peronospora effusa* R13, R19, and mock inoculated samples mapped to the spinach genome by timepoint and spinach cultivar Viroflay, or the near isogenic lines, NIL1 and NIL3

Supplemental Table 1. Potential defense-related genes of interest from R19-inoculated NIL3 spinach at 1 dpi

Supplemental Table 2. Potential defense-related genes of interest from R19-inoculated NIL3 spinach at 6 dpi

Supplemental Table 3. Enriched genes from “response to other organism” GO category in R19-inoculated NIL3 spinach at 6 dpi

Supplemental Table 4. Homologs of previously predicted R genes in spinach.

Supplemental Table 5. Differential expression in potential resistance genes from *P. effusa* R13-inoculated spinach

Supplemental Table 6. Differential expression in potential resistance genes from *P. effusa* R19-inoculated spinach

Supplemental Figure 2. Differentially expressed genes (DEGs) across *Peronospora effusa* race 13 and 19 transcriptomes

S

u

p

p

l

e

m

e

n

t

a

l

F

i

g

.

1

R

1

3

v

s

.

m

o

c

k

-

i

n

o

c

u

l

a

t

e

d

b

y

t

i

m

e

p

o

i

n

t

R

1

9

v

s

.

m

o

c

k

-

i

n

o

c

u

l

a

t

e

d

b

y

t

i

m

e

p

o

i

n

t

R

1

9

v

s

.

m

o

c

k

-

i

n

o

c

u

l

a

t

e

d

b

y

t

i

m

e

p

o

i

n

t

a

n

d

c

u

l

t

i

v

a

r

R

1

3

v

s

.

m

o

c

k

-

i

n

o

c

u

l

a

t

e

d

b

y

t

i

m

e

p

o

i

n

t

a

n

d

c

u

l

t

i

v

a

r

**S**

**u**

**p**

**p**

**l**

**e**

**m**

**e**

**n**

**t**

**a**

**l**

**F**

**i**

**g**

**.**

**1**

**.**

P

r

i

n

c

i

p

l

e

c

o

m

p

o

n

e

n

t

a

n

a

l

y

s

i

s

(

P

C

A

)

o

f

t

o

t

a

l

m

a

p

p

e

d

r

e

a

d

s

o

f

*P*

*e*

*r*

*o*

*n*

*o*

*s*

*p*

*o*

*r*

*a*

*e*

*f*

*f*

*u*

*s*

*a*

R

1

3

,

R

1

9

,

a

n

d

m

o

c

k

i

n

o

c

u

l

a

t

e

d

s

a

m

p

l

e

s

m

a

p

p

e

d

t

o

t

h

e

s

p

i

n

a

c

h

g

e

n

o

m

e

b

y

t

i

m

e

p

o

i

n

t

a

n

d

s

p

i

n

a

c

h

c

u

l

t

i

v

a

r

V

i

r

o

f

l

a

y

,

o

r

t

h

e

n

e

a

r

i

s

o

g

e

n

i

c

l

i

n

e

s

,

N

I

L

1

a

n

d

N

I

L

3

**A**

**)**

R

1

3

(

r

e

d

)

a

n

d

m

o

c

k

(

b

l

u

e

)

i

n

o

c

u

l

a

t

e

d

s

a

m

p

l

e

s

a

r

e

d

e

m

a

r

c

a

t

e

d

b

y

t

i

m

e

p

o

i

n

t

:

1

d

p

i

(

s

q

u

a

r

e

)

a

n

d

6

d

p

i

(

d

o

t

)

.

**B**

**)**

R

1

3

(

r

e

d

)

a

n

d

m

o

c

k

(

b

l

u

e

)

i

n

o

c

u

l

a

t

e

d

s

a

m

p

l

e

s

a

r

e

d

e

m

a

r

c

a

t

e

d

b

y

s

p

i

n

a

c

h

t

y

p

e

:

N

I

L

1

(

d

o

t

)

,

N

I

L

3

(

t

r

i

a

n

g

l

e

)

,

c

u

l

t

i

v

a

r

V

i

r

o

f

l

a

y

(

s

q

u

a

r

e

)

w

i

t

h

l

i

g

h

t

e

r

c

o

l

o

r

s

h

a

d

e

s

f

o

r

1

d

p

i

(

l

i

g

h

t

r

e

d

=

R

1

3

,

l

i

g

h

t

b

l

u

e

=

m

o

c

k

)

a

n

d

d

a

r

k

e

r

s

h

a

d

e

s

f

o

r

6

d

p

i

(

d

a

r

k

r

e

d

=

R

1

3

,

d

a

r

k

b

l

u

e

=

m

o

c

k

)

.

**C**

**)**

R

1

9

(

r

e

d

)

a

n

d

m

o

c

k

(

b

l

u

e

)

i

n

o

c

u

l

a

t

e

d

s

a

m

p

l

e

s

d

e

m

a

r

c

a

t

e

d

b

y

t

i

m

e

p

o

i

n

t

:

1

d

p

i

(

s

q

u

a

r

e

)

a

n

d

6

d

p

i

(

d

o

t

)

.

**D**

**)**

R

1

9

(

r

e

d

)

a

n

d

m

o

c

k

(

b

l

u

e

)

i

n

o

c

u

l

a

t

e

d

s

a

m

p

l

e

s

d

e

m

a

r

c

a

t

e

d

b

y

s

p

i

n

a

c

h

t

y

p

e

:

N

I

L

1

(

d

o

t

)

,

N

I

L

3

(

t

r

i

a

n

g

l

e

)

,

V

i

r

o

f

l

a

y

(

s

q

u

a

r

e

)

w

i

t

h

l

i

g

h

t

e

r

c

o

l

o

r

s

h

a

d

e

s

f

o

r

1

d

p

i

(

l

i

g

h

t

r

e

d

=

R

1

9

,

l

i

g

h

t

b

l

u

e

=

m

o

c

k

)

a

n

d

d

a

r

k

e

r

s

h

a

d

e

s

f

o

r

6

d

p

i

(

d

a

r

k

r

e

d

=

R

1

9

,

d

a

r

k

b

l

u

e

=

m

o

c

k

)

.

**A**

**B**

**C**

**D**

**Supplemental Table 1.** Potential defense-related genes of interest from R19-inoculated NIL3 spinach at 1 dpi

| **Gene ID** | **Description** | **Log_2_Fold-Change** |
| --- | --- | --- |
| ***Protein kinase-like*** |  |  |
| Spiol06Chr25271 | G-type lectin S-receptor-like serine/threonine-protein kinase At1g11300 | 3.00 |
| Spiol03Chr19484 | serine/threonine-protein kinase PBL27 | 2.64 |
| Spiol05Chr26812 | probably inactive leucine-rich repeat receptor-like protein kinase At5g48380 | 1.47 |
| ***P-loop containing*** |  |  |
| Spiol0329C00667 | putative disease resistance protein RGA3 | 3.52 |
| Spiol01Chr15873 | cell division cycle protein 48 homolog | -8.77 |
| Spiol03Chr17884 | DNA-binding protein SMUBP-2 | 2.27 |

**Supplemental Table 2.** Potential defense-related genes of interest from R19-inoculated NIL3 spinach at 6 dpi

| **Gene ID** | **Description** | **Log_2_fold-change** |
| --- | --- | --- |
| ***Protein kinase-like*** |  |  |
| Spiol04Chr11049 | probable LRR receptor-like serine/threonine-protein kinase At3g47570 | 10.58 |
| Spiol06Chr21950 | protein SPA1-RELATED 2-like | -3.50 |
| Spiol04Chr01380 | calcium-dependent protein kinase 26 | 1.41 |
| Spiol0084S08137 | probable leucine-rich repeat receptor-like protein kinase At5g49770 | 1.77 |
| Spiol03Chr20299 | probable LRR receptor-like serine/threonine-protein kinase At3g47570 | 3.17 |
| Spiol03Chr17794 | L-type lectin-domain containing receptor kinase VIII.2-like | 1.57 |
| ***P-loop containing*** |  |  |
| Spiol0235C00406 | cytosolic sulfotransferase 5-like | -1.62 |
| Spiol06Chr03998 | ABC transporter G family member 22 isoform X1 | -2.43 |
| Spiol04Chr09723 | ABC transporter I family member 17 | -1.80 |
| Spiol02Chr29772 | myosin-1 isoform X1 | -1.49 |

**Supplemental Table 3.** Enriched genes from “response to other organism” GO category in R19-inoculated NIL3 spinach at 6 dpi

| **Gene ID** | **Description** | **Log_2_fold-change** |
| --- | --- | --- |
| Spiol01Chr13960 | protein EARLY RESPONSIVE TO DEHYDRATION 15-like | 1.38 |
| Spiol01Chr14032 | probable pectate lyase 18 | 2.62 |
| Spiol01Chr14950 | heat shock cognate 70 kDa protein 2-like | -1.59 |
| Spiol01Chr15233 | coronatine-insensitive protein 1 | 2.01 |
| Spiol01Chr16016 | pectinesterase 2.1-like | 3.14 |
| Spiol02Chr04530 | aspartyl protease AED3 | 1.78 |
| Spiol02Chr28441 | beta-xylosidase/alpha-L-arabinofuranosidase 2-like | 1.81 |
| Spiol02Chr30479 | ferric reduction oxidase 2-like | -4.97 |
| Spiol03Chr02729 | heat shock 70 kDa protein 8 | -3.3 |
| Spiol03Chr17673 | oxalate--CoA ligase-like | -2.95 |
| Spiol03Chr17794 | L-type lectin-domain containing receptor kinase VIII.2-like | 1.57 |
| Spiol03Chr18108 | spermine synthase | -1.47 |
| Spiol03Chr19501 | superoxide dismutase [Cu-Zn] 2 | 2.13 |
| Spiol04Chr09424 | cysteine protease RD19A-like | -1.42 |
| Spiol04Chr10069 | luminal-binding protein 5-like | -4.35 |
| Spiol04Chr11001 | transcription factor HBI1-like | 1.64 |
| Spiol05Chr28227 | lysine-specific demethylase JMJ25-like | -2.21 |
| Spiol05Chr34532 | proteinase inhibitor-like | 1.62 |
| Spiol06Chr21349 | desumoylating isopeptidase 1 | -1.77 |
| Spiol06Chr22405 | probable indole-3-acetic acid-amido synthetase GH3.6 | 4.26 |
| Spiol06Chr22867 | peroxisomal acyl-coenzyme A oxidase 1-like | 1.16 |

| **Supplemental Table 4.** Homologs of previously predicted R genes in spinach | | | | |  |
| --- | --- | --- | --- | --- | --- |
| Query | Hit | %Identity | Overlap | E-value | %Gaps |
| Spo12784 | Spiol04Chr11575* | 85.68 | 98.07 | 0 | 2.82 |
|  | Spiol01Chr02529* | 71.25 | 71.35 | 0 | 3.23 |
|  | Spiol04Chr10906* | 73.15 | 71.23 | 0 | 2.24 |
|  | Spiol01Chr15405* | 69.35 | 60.24 | 0 | 3.3 |
|  | Spiol04Chr01391* | 67.97 | 41.66 | 1.27E-137 | 3.9 |
|  | Spiol03Chr02880* | 66.94 | 41.34 | 2.80E-114 | 5.43 |
| Spo12903 | Spiol03Chr02880 | 85.98 | 76.7 | 0 | 3.58 |
|  | Spiol03Chr20045* | 83.92 | 74.03 | 0 | 2.73 |
|  | Spiol04Chr01391 | 68.14 | 52.74 | 0 | 4.33 |
|  | Spiol03Chr18201* | 85.1 | 44.72 | 0 | 1.19 |
|  | Spiol01Chr15405 | 63.5 | 41.71 | 1.69E-51 | 6.19 |
| Spo12821 | Spiol03Chr02880 | 81.02 | 91.17 | 0 | 5.5 |
|  | Spiol03Chr20045 | 80.65 | 87.07 | 0 | 4.6 |
|  | Spiol03Chr18201 | 80.63 | 57.21 | 0 | 1.85 |
|  | Spiol04Chr01391 | 68.37 | 45.11 | 3.75E-170 | 3.36 |

*The eight homologs are each indicated by an asterisk. Some genes were homologs of more than one of the three previously predicted potential R genes.

**Supplemental Table 5.** Differential expression in potential resistance genes from *P. effusa* R13-inoculated spinach

| **Potential R gene homologs^A^ in Spinach v3 genome** | **Log_2_fold-change^B^** | | | | | |
| --- | --- | --- | --- | --- | --- | --- |
|  | **Vir 1dpi**  **(susceptible)** | **Vir 6dpi**  **(susceptible)** | **NIL1 1dpi**  **(resistant)** | **NIL1 6dpi**  **(resistant)** | **NIL3 1dpi**  **(susceptible)** | **NIL3 6dpi**  **(susceptible)** |
| Spiol04Chr11575 | **-1.55** | **-2.94** |  |  |  |  |
| Spiol04Chr10906 |  |  | **11.1** |  |  |  |
| Spiol01Chr02529 | **-0.95** |  | **8.07** | **-7.83** | **7.66** | **-8.9** |
| Spiol01Chr15405 |  |  |  |  |  |  |
| Spiol04Chr01391 | **-1.54** | **1.66** |  |  | **-2.44** |  |
| Spiol03Chr02880 | **0.76** | **-1.2** | **2.1** |  |  |  |
| Spiol03Chr20045 | **-0.95** |  |  |  | **3.17** |  |
| Spiol03Chr18201 |  |  |  |  |  |  |

^A^ Potential resistance gene homologs to either Spo12784 or Spo12903 from She et. al. 2018 or to Spo12821 from Gao et. al. 2022.

^B^ All a log_2_fold-change values shown have a P-values of 0.05 or lower.

**Supplemental Table 6.** Differential expression in potential resistance genes from *P. effusa* R19-inoculated spinach

|  | **Log_2_fold-change^B^** | | | | | |
| --- | --- | --- | --- | --- | --- | --- |
| **Potential R gene homologs^A^ in Spinach v3 genome** | **Vir 1dpi**  **(susceptible)** | **Vir 6dpi**  **(susceptible)** | **NIL1 1dpi**  **(susceptible)** | **NIL1 6dpi**  **(susceptible)** | **NIL3 1dpi**  **(resistant)** | **NIL3 6dpi**  **(resistant)** |
| Spiol04Chr11575 | **-2.6** | **-4.72** |  |  | **7.37** |  |
| Spiol04Chr10906 | **-0.78** |  | **8.17** | **1.73** |  |  |
| Spiol01Chr02529 | **-1.21** |  | **7.08** |  | **7.06** |  |
| Spiol01Chr15405 |  |  |  |  |  |  |
| Spiol04Chr01391 |  |  |  |  |  |  |
| Spiol03Chr02880 | **-0.72** | **-1.18** |  |  |  |  |
| Spiol03Chr20045 | **-0.74** |  |  |  |  |  |
| Spiol03Chr18201 |  | **1.38** |  | **5.37** |  |  |

^A^ Potential resistance gene homologs to either Spo12784 or Spo12903 from She et. al. 2018 or to Spo12821 from Gao et. al. 2022.

^B^ All a log_2_fold-change values shown have a P-values of 0.05 or lower.
